# Editorial: Recent Advances in Animal Cognition and Ethology

**DOI:** 10.3390/ani13182890

**Published:** 2023-09-12

**Authors:** Cédric Sueur, Marie Pelé

**Affiliations:** 1 IPHC, Université de Strasbourg, CNRS, UMR7178, 67087 Strasbourg, France; 2 Institut Universitaire de France, 75005 Paris, France; 3 ANTHROPO-LAB, ETHICS EA 7446, Université Catholique de Lille, 59000 Lille, France; marie.pele@univ-catholille.fr

## 1. Introduction

Animal cognition and ethology, the scientific study of animal behaviour, have long captivated the human imagination. From the intricate social structures of ant colonies [1] to the astonishing problem-solving abilities of octopuses [2], the realm of animal cognition and ethology offers a glimpse into the remarkable capacities and complexities of non-human minds. This field of research has undergone a transformative journey, enriched by technological innovations and a profound shift in perspective that acknowledges animals as active agents in their own right [3]. The past few decades have witnessed a remarkable surge in the exploration of animal cognition and ethology, primarily driven by technological advancements that have expanded the boundaries of what we can observe and comprehend. Researchers have harnessed the power of drones [4,5], artificial intelligence [6,7,8], bio-loggers [9,10] and acoustic monitoring devices [11,12] to unravel the mysteries of animal behaviour in ways that were once unimaginable. These tools have enabled us to enter the natural habitats of animals, providing an unprecedented window into their lives and their cognition [3]. Crucially, the paradigm in animal cognition and ethology has shifted from a purely observational stance to one that recognises the animal agency [13]. Researchers increasingly consider how animals collaborate and interact with their environments, leading to innovative ethical studies that transcend traditional laboratory settings. Ethologists now venture into the field, adapting their protocols for ethical research and to accommodate the agency of animals in their natural habitats [14,15,16,17]. This special issue ‘Recent Advances in Animal Cognition and Ethology’, showcases a diverse array of scientific papers that exemplify this transformative journey. We invite readers to explore the remarkable research presented in the following pages, each paper contributing a vital piece to the ever-expanding puzzle of understanding animal minds and behaviour. Together, these studies redefine our relationship with non-human animals. We introduced the papers of this special issue according to three themes: Advances in ethology and cognition, the role of cutting-edge technologies and advances in animal research ethics.

## 2. Advances in Ethology and Cognition

The ‘GeoDanceHive’ [18] offers a unique glimpse into honeybee communication by recording their dances in natural settings. This innovative tool promises to revolutionise the study of honeybee behaviour without disrupting hive activities. Similarly, researchers used artificial intelligence to analyse non-figurative drawings created by an orangutan [19], revealing subtle seasonal variations and emphasising the potential of AI in objectively interpreting animal-produced art. Other apes, chimpanzees, were the focus of one study, which delved into their numerical abilities [20]. This research challenged our understanding of numerical cognition in primates, demonstrating that sequencing Arabic numerals is more challenging for chimpanzees than previously thought, shedding light on global-local processing differences between humans and chimpanzees. Communication in animals, an essential aspect of ethology, was a common theme of several papers. The impact of mining noise on marmoset vocalisations [21] raised concerns about human activities disrupting the acoustic communication patterns of these primates. In contrast, AI-driven analysis helped detect indris’ songs in passive acoustic recordings [22], providing valuable insights into the vocal behaviours of this endangered species. Two studies explored the social dynamics and cultural influences on animal behaviour. In Nepal, an etho-ethnographic study examined the social behaviour of domestic yaks, demonstrating the role of cultural values in herd cohesion [23]. In a separate investigation, researchers observed leave-taking behaviour in wild chacma baboons [24], challenging the notion that this behaviour is unique to humans and suggesting a deep evolutionary history. Several studies used innovative approaches to understand complex animal societies and networks. A simulation of trophallactic networks in ants [25] revealed the significance of interindividual variability and division of labour in shaping efficient trophallactic networks, enhancing our understanding of self-organisation in ant colonies. Drones were utilised to study multilevel animal societies [26], providing insights into the positioning patterns and behavioural propagation mechanisms in such groups. Finally, a review paper [27] highlighted the application of information theory in the analysis of animal behavioural sequences. This approach proved effective in decoding stereotypic behavioural sequences and revealing the existence of a symbolic ‘language’ in leader-scouting ant species, offering new avenues for experimental studies of animal behaviour and communication. Collectively, these studies represent a significant leap forward in our comprehension of animal cognition and ethology. They underscore the importance of embracing technological innovations and recognising the agency of animals in their natural environments. These insights not only deepen our understanding of the animal kingdom but also have profound implications for conservation and ethical considerations in our interactions with other species.

## 3. The Role of Cutting-Edge Technologies

In recent years, a wave of technological innovation has revolutionised the field of ethology and animal cognition, propelling our understanding of non-human minds and behaviours to new heights. The eleven groundbreaking studies of this special issue exemplify the transformative impact of these cutting-edge technologies. One notable innovation is the ‘GeoDanceHive’, a hive prototype equipped with sensors and recording devices that enable real-time observation of honeybee dances in their natural environment [18]. This non-invasive tool avoids disrupting hive activities, offering unprecedented insights into resource communication. Advancements in touchscreen technology played a pivotal role in assessing chimpanzee numerical abilities [20] and other capacities [28]. These controlled experiments revealed distinctions in numerical cognition between humans and chimpanzees, underscoring the potential of touchscreen-based tasks. Similarly, a computer-controlled touchscreen system offered a novel approach to examine visual discrimination abilities in Garrano horses [29]. This technology facilitated controlled experiments without the potential bias introduced by human interactions. Passive acoustic monitoring devices were employed to investigate the impact of mining noise on marmoset vocalisations [21]. This non-intrusive technology allowed researchers to study how anthropogenic noise affects animal communication patterns without direct human interference. Artificial intelligence, specifically convolutional neural networks, emerged as a powerful tool for the automated detection of indris’ songs in passive acoustic recordings [22]. This deep learning approach expedited the analysis of extensive datasets, enhancing our understanding of primate vocalisations. Information theory, based on Shannon entropy and Kolmogorov complexity, became a valuable tool for analysing and comparing animal behavioural sequences [27]. It proved effective in uncovering complex natural behaviours. Video footage and multivariate analysis were instrumental in identifying behavioural cues associated with leave-taking in wild chacma baboons [24]. These advanced analysis techniques shed light on social behaviours in non-human populations. Simulation models, incorporating interindividual variability and division of labour, provided insights into the trophallactic networks of ants, unravelling the rules governing food distribution in complex societies [25]. This might be extended to simpler insect models as Drosophila [30,31]. For bigger social species as mammals, drones played a pivotal role in studying complex multilevel animal societies, enabling the identification and tracking of individuals within these groups [26]. This technology unveiled spatial positioning patterns and behavioural propagation mechanisms in complex social settings. Collectively, these studies highlight the transformative power of technology in advancing ethology and animal cognition research. From non-invasive monitoring devices to deep learning algorithms, these innovations have expanded our capacity to explore the cognitive abilities and behaviours of animals, offering unprecedented insights into their natural worlds. As technology continues to evolve, so too will our understanding of the complex lives and minds of our fellow creatures.

## 4. Advances in Animal Research Ethics

Ethical considerations are paramount in animal research [32,33], and the eleven studies of the special issue demonstrate a commitment to the humane treatment and ethical handling of animals. These studies have not only advanced our understanding of non-human minds and behaviours but have done so while prioritising the welfare and ethical treatment of the animals involved. In the study involving honeybees and the GeoDanceHive [18], researchers designed a non-invasive tool that allowed the continuous recording of honeybee dances in their natural environment. Importantly, this innovative technology ensured that the activities of the bees were not disrupted, and the welfare of the hive was maintained. This approach exemplifies a dedication to studying animal behaviour while minimising intrusion [34,35]. The research on chimpanzee numerical abilities [20] employed touchscreen-based experiments, which are non-invasive and enable controlled assessments of cognitive abilities. This use of technology allowed researchers to collect data without causing harm or stress to the chimpanzees, aligning with ethical research practices. Similarly, the study on Garrano horses’ visual discrimination [29] utilised a computer-controlled touchscreen system, offering a non-invasive method for assessing the horses’ cognitive abilities. This approach ensures that the horses’ welfare is upheld while contributing valuable insights into their visual discrimination skills. Touchscreen can now be used with a large panel of species [36,37] and into the wild [3,38]. The passive acoustic monitoring of marmosets in a noisy mining environment prioritised the animals’ well-being [21]. By using non-intrusive acoustic monitoring devices, researchers could investigate the impact of anthropogenic noise on marmoset vocalisations without directly interfering with the animals’ natural behaviour. Moreover, the automated detection of indris’ songs through artificial intelligence [22] showcased a non-invasive approach to studying primate vocalisations. This technology allowed researchers to analyse extensive datasets without any harm to the indris or their habitat. Leave-taking behaviour in wild chacma baboons [24] was investigated using video footage and multivariate analysis, which do not require direct contact with the animals as for the use of drones in studying multilevel animal societies, while groundbreaking, does not compromise animal welfare. Drones provide a non-invasive means of observing and tracking animals within their natural habitats [26]. Simulation models employed in the trophallactic network study of ants do not involve real animals, thus eliminating ethical concerns related to animal welfare. These models allowed researchers to explore complex behaviours without impacting living organisms. In summary, these studies in ethology and animal cognition demonstrate a commitment to ethical research practices. Researchers have leveraged innovative but non-invasive methodologies to advance our understanding of animal behaviour while ensuring the welfare and ethical treatment of the animals involved. These efforts underscore the importance of ethical considerations in animal research and contribute to responsible and humane scientific inquiry.

## 5. Conclusions

The collective body of research presented in this special issue represents a significant leap forward in our understanding of animal cognition and ethology. Through innovative technologies and ethical research practices, these studies have illuminated the intricate workings of non-human minds and behaviours, offering profound insights into the natural world. These studies have harnessed cutting-edge tools to explore the cognitive capabilities and behavioural intricacies of a diverse array of animal species. Moreover, ethical considerations have been at the forefront of these investigations, with researchers prioritising the welfare and well-being of the animals involved. Non-invasive methods have allowed for the study of animal behaviour without causing harm or stress to the subjects. This is the future of animal research.
